# IL-26: An Emerging Proinflammatory Member of the IL-10 Cytokine Family with Multifaceted Actions in Antiviral, Antimicrobial, and Autoimmune Responses

**DOI:** 10.1371/journal.ppat.1005624

**Published:** 2016-06-23

**Authors:** Emmanuel Stephen-Victor, Helmut Fickenscher, Jagadeesh Bayry

**Affiliations:** 1 Institut National de la Santé et de la Recherche Médicale Unité 1138, Paris, France; 2 Centre de Recherche des Cordeliers, Equipe, Immunopathologie et immuno-intervention thérapeutique, Paris, France; 3 Sorbonne Universités, Université Pierre et Marie Curie, Paris 06, UMR S 1138, Paris, France; 4 Institute for Infection Medicine, Christian Albrecht University of Kiel and University Medical Center Schleswig-Holstein, Kiel, Germany; 5 Université Paris Descartes, Sorbonne Paris Cité, UMR S 1138, Paris, France; McGill University, CANADA

Cytokines are small proteins that mediate signaling in immune and nonimmune cells, resulting in the modulation of cellular differentiation and activation. These functions are not only important for inflammation but also for antimicrobial responses. Additionally, inflammatory cytokines such as IL-1β and IL-17 can directly interact with microbes and promote their growth [1,2]. In this context, IL-26, an emerging member of IL-10 family cytokines, stands distinct as it exerts antimicrobial response not only by priming various innate immune cells and modulating antiviral responses but also by eliciting direct microbicidal action through affecting the formation of membrane pores.

## IL-10 Family Cytokines and Antimicrobial Responses

IL-10 family members play an important role in tissue remodeling and wound healing following infection and inflammation [3]. Growing evidence also suggests an important role for IL-10 family cytokines in mediating and regulating immune response to pathogens. To date, nine cytokines have been categorized into the IL-10 clan, which includes IL-10, the IL-20 subfamily cytokines (IL-19, IL-20, IL-22, IL-24, IL-26), and the distantly related type III interferons (IFN) IL-28A, IL-28B, and IL-29.

**Fig 1:**
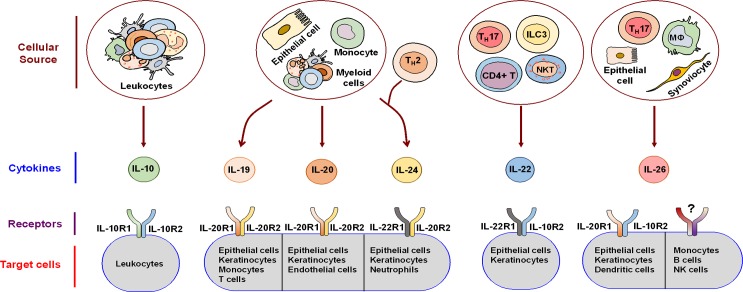
IL-10 family cytokines: source, receptors, and target cells. IL-10 family cytokines are produced by both immune and non-immune cells and signal through heterodimeric receptors expressed on diverse target cells. However, IL-26 might also mediate heterodimeric IL-20R1/IL-10R2 receptor-independent signaling. This scheme was drawn in part by using pictures from Servier Medical Art. NKT, Natural killer T cells; ILC3, Group 3 innate lymphoid cell; MΦ, Macrophage.

IL-10 is a potent anti-inflammatory cytokine that signals through the heterodimeric receptor formed by IL-10R1 and IL-10R2 (Fig 1). By suppressing pro-inflammatory responses elicited by various immune cells, IL-10 limits tissue damage and immunopathology caused during infections [3]. On the other hand, IL-28A, IL-29B, and IL-29 synergize with type I IFNs to support antiviral responses [8].

The IL-20 subfamily cytokines, in general, are vital for innate defense mechanisms at epithelial surfaces. IL-22, the most studied IL-20 subfamily cytokine, regulates epithelial homeostasis and induces antimicrobial agents and β-defensins. IL-22 is produced by lymphocytes and signals through IL-10R2/IL-22R1 heterodimer (Fig 1). IL-22 controls gut microbiota and is involved in the defense against mucosal infections, including those caused by *Candida* and *Klebsiella pneumonia* [4,5]. IL-19, IL-20, and IL-24, collectively known as IL-20 receptor (IL-20R) cytokines, are produced mainly by epithelial cells and myeloid cells and signal through type I IL-20R (IL-20R1 and IL-20R2). IL-20 and IL-24 additionally signal through type II IL-20R (IL-20R2 and IL-22R1) as well. IL-20R cytokines are critical for tissue homeostasis, epithelial proliferation, and antimicrobial peptide secretion. Recent evidence in a murine model further suggests that IL-20RB-mediated signaling by IL-19 and IL-20 at skin is immunosuppressive and exacerbates cutaneous *Staphylococcus aureus* infection by curtailing IL-1β production and Th17 pathways [6]. Conversely, exogenous administration of IL-24 was reported to modulate IFN-γ responses and neutrophil functions in *Salmonella* infections in vitro and in vivo [7]. The paucity of data warrants further investigations into the precise role of IL-20R cytokines in various infections.

## IL-26: An Emerging Member of IL-10 Family Cytokines

IL-26 is an emerging member of IL-10 family cytokines. It was initially named as AK155 following its identification in herpesvirus saimiri-transformed human T cells [9]. Epithelial cells and immune cells, including alveolar macrophages, Th1 and Th17 cells, NK cells, and macrophage-like synoviocytes, are predominant sources of IL-26. The *IL-26* gene is located on chromosome 12q15 and is flanked by genes encoding for two important cytokines, IL-22 and IFN-γ. Secreted IL-26 is a 19-kDa protein containing 171 amino acids with approximately 25% homology and 47% similarity to human IL-10. The protein is largely cationic (~20%), resulting in a positive charge with a calculated isoelectric point of 10.4. IL-26 comprises six helices with a capacity to form dimers and higher-order multimers [9]. IL-26 signals via the heterodimeric IL-20R1/IL-10R2 receptor (Fig 1) and induces Janus kinase-signal transducer and activator of transcription (JAK-STAT) activation, resulting in STAT1 and STAT3 phosphorylation [10,11]. Current evidence suggests that IL-26 recognizes IL-20R1 directly, whereas IL-10R2 helps in the proper assembly of functional IL-26 receptor complex. Although the distribution of IL-10R2 is broad, only a subset of cells, particularly epithelial cells and keratinocytes, expresses IL-20R1 [11]. A recent report shows that myeloid cells, like monocyte-derived dendritic cells, also express mRNA for IL-20R1 [12]. This restricted expression pattern of IL-20R1 limits the action of IL-26.

IL-26 also mediates IL-26 receptor-independent signaling as reported in monocytes and B cells [13–15]. This interaction might be mediated via a hitherto unidentified surface receptor or possibly due to the highly cationic nature of IL-26 that facilitates binding to several glycosaminoglycans, including heparin and heparan sulphate, on the cell surfaces [11]. Many functions of IL-26 have only recently been elucidated.

## IL-26 Has Diverse Antiviral and Antimicrobial Actions

As expression of IL-20RA, the key subunit of IL-26R that mediates IL-26 signaling, is restricted to skin, intestine, and lungs, it is thought that IL-26 promotes defense mechanisms at mucosal surfaces by bridging immune cells and epithelia. In fact, antimicrobial functions of IL-20 subfamily cytokines, including Th17-derived IL-22, are mediated mainly through the induction of various antimicrobial peptides (i.e., S100A7, S100A8, and S100A9, comprising a group of highly conserved proteins functioning as an immediate line of defense against diverse pathogens) in epithelial cells. However, IL-26 is unique because it does not promote the production of antimicrobial peptides, instead acting as an antimicrobial protein itself.

**Fig 2:**
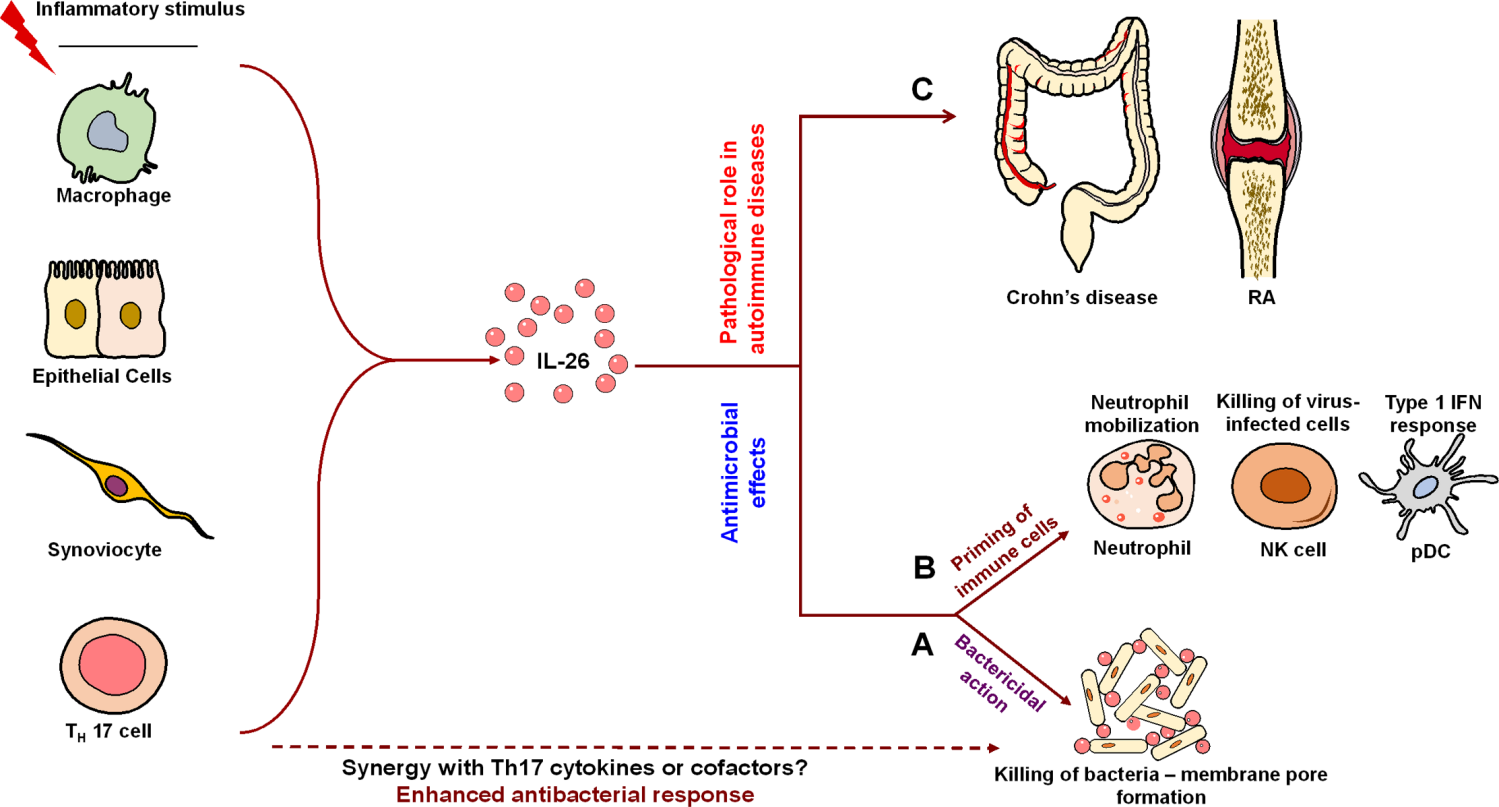
Multifaceted actions of IL-26 in antiviral, antimicrobial, and autoimmune responses. Upon activation, various immune cells secrete IL-26. IL-26 exerts antiviral and antimicrobial actions through dual action by (A) direct killing of bacteria by forming membrane pores and (B) by priming immune cells, such as neutrophils, NK cells, and plasmacytoid dendritic cells (pDCs). Other Th17 cell-derived molecules might act in synergy with IL-26 to enhance this direct killing of bacteria. (C) IL-26 response requires tight regulation as increased expression of IL-26 has been reported in several autoimmune and inflammatory diseases, including Crohn’s disease and rheumatoid arthritis (RA). This scheme was drawn in part by using pictures from Servier Medical Art.

IL-26 exhibits priming effects on various immune cells in order to boost antiviral and antimicrobial responses. IL-26 induces TNF-related apoptosis-inducing ligand (TRAIL) on human NK cells that kill hepatitis C virus-infected hepatocytes [16]. Furthermore, IL-26 derived from CD68^+^ alveolar macrophages and Th17 cells propel antimicrobial responses by priming the recruitment of neutrophils towards bacteria and assembled effector immune cells at the lungs, and by triggering the production of plasmacytoid dendritic cell (pDC)-derived IFN-α (Fig 2), respectively [17,18].

Recent data provide new dimensions to IL-26 functions. Sequence analysis and three-dimensional modeling revealed the cationic amphipathic nature of human IL-26 along with an ability to form multimers, which are similar to other antimicrobial peptides such as defensins. It was reported that human Th17 cell-derived IL-26 mediates protective immunity by direct microbicidal action due to its functional similarity to naturally occurring antimicrobial peptides. When examined, recombinant human (rh) IL-26 inhibited the growth of gram-negative bacteria, such as *Pseudomonas aeruginosa*, *Escherichia coli*, and *K*. *pneumonia*, and gram-positive bacteria like *S*. *aureus* by direct bactericidal action. The median minimum inhibitory concentration values were in the range of 8.6 to 18.6 μM. IL-26-mediated killing was attributed to the formation of membrane pores and blebs on the surface of bacteria causing membrane disruption and leakage of cytosolic contents (Fig 2) [18]. The bactericidal action of IL-26 was also facilitated in part by its capability to bind lipopolysaccharide and lipoteichoic acid of gram-negative and -positive bacteria with dissociation constants (*K*_*D*_) of 40.5 ± 4.95 nM and 20.91 ± 2.84 nM, respectively. Local IL-26 concentrations might be considerably increased due to the cationic nature of IL-26 and its affinity to glucoaminoglycans on the cell surfaces [11,15).

IL-26 displays differential abilities in modulating the rate of epithelial and fibroblast cell infections by enveloped viruses. This IL-26R-independent effect was proposed to be largely due to the cationic nature of IL-26, which might accelerate either the binding to or repulsion of viruses from the cells [15].

## IL-26 Response Requires Tight Regulation during Infection

It is critical that IL-26 production and signaling be tightly regulated. Chronic infections and their pathological consequences as observed in human lymphatic filariasis are reported to be associated with increased IL-26 expression [19]. IL-26 is up-regulated in the skin and colonic lesions of psoriatic and inflammatory bowel disease patients [20–22]. By binding to its receptors expressed on intestinal epithelial cells, IL-26 inhibits their proliferation and concomitantly induces the production of pro-inflammatory cytokines TNF-α and IL-8 [11,21]. Moreover, increased expression of IL-26 in colonic RORγt-expressing Th17 cells is correlated with the pathogenesis of Crohn’s disease (Fig 2) [21]. Similarly, in rheumatoid arthritis, synoviolin^+^ fibroblast-like synoviocytes and CD68^+^ macrophage-like synoviocytes constantly secrete IL-26 [13]. Because the aforementioned pathologies have often been suspected to be of infectious etiology [23], these data suggest that once the infection is cleared, IL-26 should be switched-off in order to prevent secondary pathologies. Alternatively, as human IL-26, IFN-γ, and IL-22 are transcribed in the same orientation with related expression patterns and partially common regulatory elements [9,24], it is likely that uncontrolled Th1 and Th17 responses are associated with the IL-26 response in these pathologies.

## Therapeutic Use of IL-26 for Bacterial Infections: Future Directions

The newly discovered potent antibacterial action of IL-26 raises the possibility of its therapeutic use for diverse pathogens. However, several key questions remain to be addressed.

First, commensal bacteria were resistant to IL-26-mediated killing [18]. This differential killing suggests that IL-26 could be targeting pathogenic factors that might otherwise be absent in commensals. Identifying the molecular basis underlying the interaction of IL-26 with bacterial components ought to shed light on its specificity and reveal antigenic components that could be exploited for the therapeutic induction of IL-26.

Second, rhIL-26 produced in a prokaryotic expression system was relatively less efficient in killing the bacteria when compared to IL-26 in Th17 cell supernatants. Differences in post-translational modifications between recombinant and Th17-derived natural IL-26 could be the possible explanations. Comparing antimicrobial activities of rhIL-26 produced in prokaryotic and mammalian expression systems could address this issue. Alternatively, cooperation with other Th17 molecules that act in synergy with IL-26 might not be ruled out and requires further investigations.

Third, the absence of IL-26 in mice has greatly hindered our progress in understanding the antimicrobial functions of IL-26, its regulation, and therapeutic applications for various bacterial diseases. Generating IL-26 transgenic mice might be the solution moving forward.

Nevertheless, the potent bactericidal action of IL-26 raises the possibility of its therapeutic use against selected pathogens, particularly when confronted with antibiotic-resistant bacterial strains. As microbicidal action of IL-26 is independent of other components of the immune system, it also offers a therapeutic option for immunocompromised individuals either alone or in combination with anti-infective immunoglobulins [25,26].
